# Optimization the Stab Resistance and Flexibility of Ultra-High Molecular Weight Polyethylene Knitted Structure Fabric with Response Surface Method

**DOI:** 10.3390/polym15234509

**Published:** 2023-11-23

**Authors:** Xuliang Yu, Ting Su, Xinhua Liang, Honglian Cong

**Affiliations:** Engineering Research Center of Knitting Technology, Jiangnan University, Ministry of Education, Wuxi 214122, China; yuxul0214@163.com (X.Y.); m15538390368@163.com (T.S.); liangxh18861852560@163.com (X.L.)

**Keywords:** UHMWPE, knitted material, yarn shear resistance, stab resistance, flexibility

## Abstract

At present, the challenging issue of the compatibility between stab resistance and flexibility of materials frequently appears. Thus, this study proposes a novel method to enhance the comprehensive performance of the material matrix with stab resistance. Based on the stab-resistant mechanism analysis of the textile matrix, the influence of four factors on the performance of ultra-high molecular weight polyethylene (UHMWPE) knitted fabric was discussed. And, the optimal process conditions of material for achieving high stab resistance and high flexibility were obtained by the response surface method. A series of experiments proved that among all factors, the fabric structure had the greatest influence on the flexible stab-resistant knitted material. Following that, the thickness of the yarn also plays a significant role. Under the optimal process conditions, the stab peak force of the knitted material was promoted to 52.450 N, and the flexibility was enhanced to 93.6%. Meanwhile, through comparison with products that have undergone the same treatment, there was little difference in stab resistance but significantly improved flexibility. It achieves the initial stab resistance and comfortable wearing softness of the fabric through process optimization. This improvement in overall performance of the textile matrix enables further enhancement treatments.

## 1. Introduction

Some personnel engaged in special work, such as those in military and public security, are often equipped with stab-resistant clothing, to protect the human torso and internal organs from injury. At the same time, the rapid innovation momentum is revealed on the stab-resistant clothing. It has been updated from hard stab-resistant clothing [1] to soft stab-resistant clothing made of high-performance fibers. Additionally, the latter has the characteristics of both protection and softness [2,3,4], which is deeply loved by the audience. Among them, ultra-high molecular weight polyethylene (UHMWPE) is one of the most commonly used raw materials in stab-resistant products as a leader in high-performance fibers.

**Figure 1:**
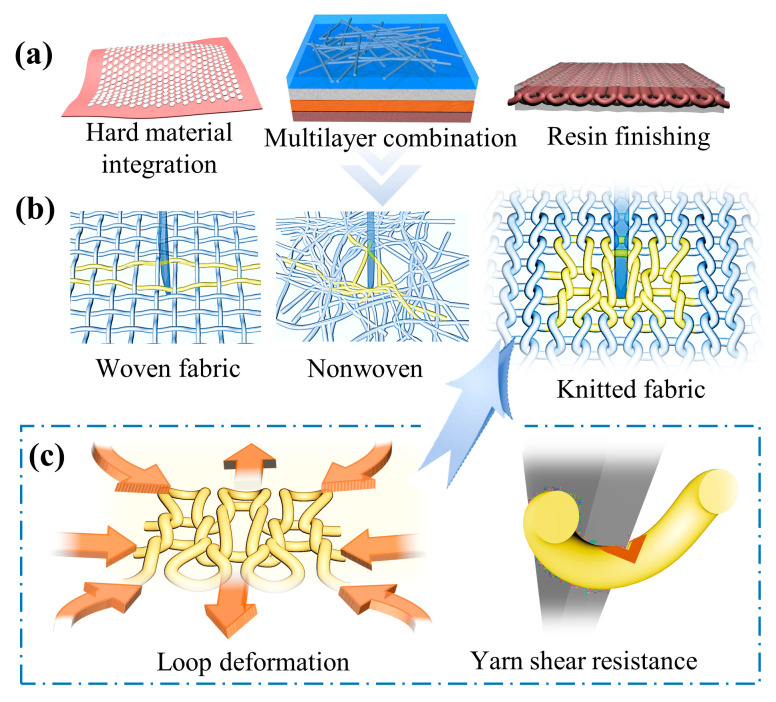
Stab-resistant mechanism of fabric structures. (**a**) Different matrix strengthening treatment methods; (**b**) simulation of three fabric structures; (**c**) analysis of the knitted loop structure stab-resistant mechanism.

Besides, UHMWPE is widely adopted in the field of flexible protective textiles and some high-tech industries due to its high breaking strength, large modulus, and low density [5,6]. However, the stab-resistant fabric made solely of UHMWPE fiber cannot meet the protection needs with perfect grades. Most research teams mainly study the combination of resin, ceramic, or shear thickening fluid with the matrix [7,8,9,10], as well as the multi-layer superposition of different materials [11], to improve the stab-resistant effect of the product, as shown in Figure 1a. For example, Mao et al. [12] integrated an epoxy resin protection block onto a soft fabric substrate to obtain a flexible, stab-resistant, and wear-resistant composite fabric. Rao et al. [13] developed a flexible stab-resistant material for personal protective equipment by laminating multi-component yarns and UHMWPE with different layers. Gurgen et al. [14] designed silicon dioxide, polyethylene glycol (PEG)-based STFs, and silicon carbide (SiC) with different particle sizes to obtain multiphase STFs. It can further improve the effect of fabric stab resistance. In summary, the latter compounding process are of more concern to researchers. Although, the protection standard of the stab-resistant materials can be achieved by the above preparation methods, and some products also have other functions such as light and flexible, breathable and comfortable, the single-layer original textile still maintains a low level and has unqualified stab resistance [15,16,17,18]. Meanwhile, other problems will also be caused by using the above process, such as instability, heat resistance, softness, thickness, brittleness, and so on [19,20,21]. The difficulty of weaving the stab-resistant material will be reduced, and the convenience of sewing the garment will be improved, if we primarily rely on the matrix textile without adding reinforcement materials. In addition, the breathability, softness, and other wear properties of the above-mentioned stab-resistant materials are certainly far less than that of the matrix textile [22,23,24,25]. Without doubt, the stab-resistant materials are required to be flexible and stab-resistant at the same time, especially when applied to some special protective clothing. However, the matrix fabric is prone to lose its soft and flexible properties when improving its stab resistance [26,27]. Therefore, it is still a challenge to improve the stab-resistant performance while maintaining the original flexibility of the stab-resistant fabric.

Currently, the matrix textiles used in stab-resistant materials are mainly divided into woven fabric, nonwoven, and knitted fabric [28,29,30], as shown in Figure 1b,c. The interweaving points between the yarn in woven plain structure fabrics and the nonwoven material are relatively unconstrained. This causes the yarn to slip easily, making the fabric lose its main stab-resistant effectiveness [31,32]. However, the knitted structure is composed of yarns interlooping and interlocking with each other, whether warp knitted or weft knitted, somewhat similar to ancient scale armor. As a result, there are a large number of entanglement points between the yarns, which gives knitted structures an unparalleled advantage over woven and nonwoven fabrics [33,34]. So, when a blade pierces a knitted fabric, the loop at the point of penetration quickly gathers the surrounding yarns to provide protection due to the abundant entanglements and connections. Specifically, the loop arc is first extended to both ends by the squeeze of the piercing blade, followed by the transfer of the loop sinking arc. Then, as the blade deepens, the yarn is continually pulled, causing the surrounding loop to pile up and squeeze around the blade. At this juncture, the friction resistance of the loop structure reaches a peak on the blade. Besides, the deformation ability of the loops can be regulated to elevate the stab-resistant effect of the knitted fabric through various means, such as altering the interloped manner of yarns by changing the fabric structure. Immediately after the loop deformation, the residual energy of the tool puncture will be absorbed by the method of yarn shearing, friction heat generation, etc., to achieve the stab-resistant effect of the knitted fabric. It can be realized the knitted loop structure greatly exerts the characteristics of high-performance fiber and absorbs large impact kinetic energy through the mechanism of loop deformation. In addition, the knitted loop structure is widely used for its excellent properties such as air permeability and softness. Therefore, the research on the optimization of the stab resistance and flexibility of UHMWPE matrix with the knitted structure is particularly important, although it is basic.

In this paper, the knitted fabric, the woven fabric, and the nonwoven were simulated and compared first, all of which were matrix textile structures commonly used in stab-resistant materials. Then, the advantages of knitting structure on stab-resistant properties were explored to further determine the influencing factors on stab-resistant and soft properties of knitted fabrics. Through the method of single-factor design, the quasi-static stab and bending stiffness experiment of knitted fabrics were carried out under different influence factors. The four factors are yarn specifications factor, yarn content factor, fabric stitch density factor, and structure factor. In the end, the response surface method (RSM) was applied to the above factors to obtain the optimal process. It is noted the response surface method is to fit the functional relationship between factors and response values with the multiple quadratic regression equation obtained from the experimental scheme. Whereafter, the optimal process combination can be accurately and reliably predicted by analyzing the regression equation. The research mentioned above has rarely been covered in previous reports. In particular, the optimization process of UHMWPE knitted fabric was calculated based on the response surface method. It makes the comprehensive performance of the stab resistance and flexibility of stab-resistant materials most excellent, which is more suitable for the subsequent process, and also directly applicable to the protection products.

## 2. Materials and Methods

### 2.1. Material Preparation

According to the above analysis, the main factors affecting the stab resistance of the knitted material are the deformation effect of the loop structure and the shear resistance characteristics of the yarn. In addition, other influencing factors are not considered in this experiment due to their small energy fluctuations. Meanwhile, it can be imagined the deformation effect of the loop is related to the stitch density, the organizational structure, and the raw material of the knitting process [35]. In theory, the tighter the stitch density, the more complicated the fabric structure, and the less the yarn stretches, the more difficult it is to deform the loop. Among them, the tensile effect of the yarn is controlled by the blending ratio of UHMWPE and 600 D high-elastic polyester yarn. In addition, some parameters are not included in the factors that directly affect the stab resistance of the fabric, such as thickness and areal density. Apart from that, the shear resistance and tensile effect of UHMWPE yarns are mainly affected by the thickness of their specifications [36]. Certainly, the effect of twisting, entanglement, and wrapping on the performance of UHMWPE yarn cannot be denied, but these factors are not the most critical. Therefore, in this experiment, different specifications of yarns are selected as another factor affecting the stab-resistant properties of fabrics to optimize the stab-resistant and flexible properties of fabrics.

Furthermore, it is fully considered too thick yarn is not suitable for use in clothing. So, the polyethylene yarn of 200–1000 D specification (Jiangsu Jiuzhou Xingji New Material Co., Ltd., Nantong, China), and its supported 7.2 E double needle computerized flat knitting machine (CMS 530 Karl Mayer, Obertshausen, Germany) were adopted for the development of stab-resistant materials. Currently, the knitted matrix available on the market and referred to in the literature is still mainly based on the interlock stitch, and then varying the number of internal loop tuck connections to achieve the protective function of the knitted products [37,38]. Meanwhile, to ensure the stab-resistant performance of the fabric, the fabric is required to be designed with a certain thickness. Therefore, the method of changing the number of tuck structures in the interlock fabric was accepted to boost the yarn content inside the fabric. A 1 × 3 structure indicated there was 1 row tuck connected in the 3 rows minimum cycle. This readily modifies fabric thickness, areal density, and even fabric tightness and stab resistance. Moreover, the stitch density of this experiment varied ±5 floating values from the base value. And, the yarn blending ratio showed the proportion of UHMWPE yarn in the total yarn content of the fabric.

### 2.2. Test Method

The shear resistance and tensile effect of different specifications of UHMWPE yarns were tested, firstly. Then, the single-factor experimental design method was adopted to analyze four groups of the factor, and each group had 5 different levels. In quick succession, the central value range of response surface method for the stab resistance and flexibility was determined by the test results. Additionally, it was required all test samples be placed in a test environment at 25 °C (±2 °C) temperature and 65% (±5%) relative humidity for 24 h. Furthermore, all tests conducted in this study were carried out in standard testing conditions.

**Figure 2:**
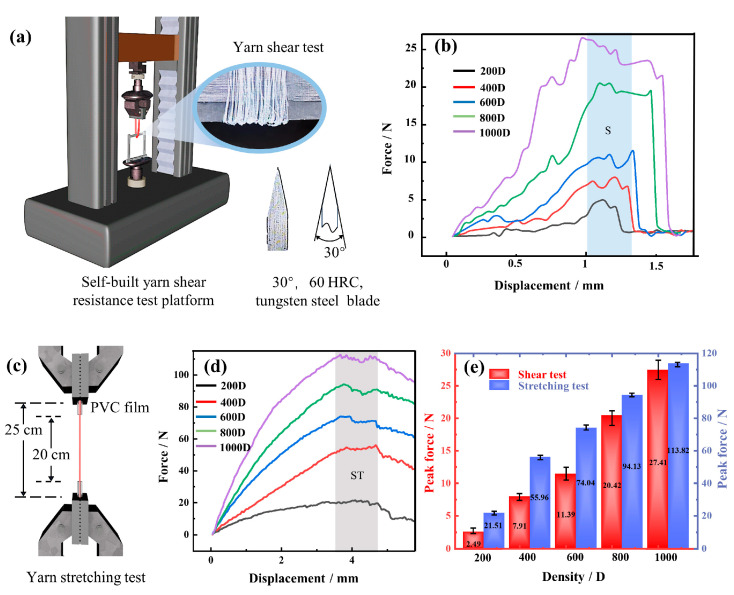
Shear resistant force and breaking strength of UHMWPE. (**a**) Model of yarn shear test platform; (**b**) shear resistant force-displacement of yarn with different specifications; (**c**) yarn stretching test; (**d**) stretch force-displacement of yarn with different specifications; (**e**) peak shear force and peak stretch force of yarn with different specifications.

At present, there is no special instrument or standard for yarn shear testing. As can be seen in Figure 2a, a fixture and a blade are installed at the lower end of the universal tensile testing machine (E43.504, MTS Systems (China) Co., Ltd., Shanghai, China) to establish a test platform for yarn shear resistance. At the start, both ends of the yarn with a test length of 20 cm are clamped on the upper fixture, while it goes around the lower end of the blade. The blade is made of 60 HRC tungsten steel with a size of 60 × 25 × 2 mm^3^, and its edge is a 30° symmetric blade. Simultaneously, the blade is placed on the fixed clip and parallel to the table. Then, different specifications of UHMWPE yarns are stretched at a constant speed of 20 mm/min to test the shear resistance.

According to the test standard of GB/T 7690. 3-2013 [39], the yarn tensile test is also carried out on the E43.504 MTS machine (MTS Systems (China) Co., Ltd., Shanghai, China). In Figure 2c, to prevent the yarn from slipping with the fixture or breaking at the jaws, both ends of UHMWPE yarn with an initial length of 25 cm are wrapped by 2.5 cm PVC film. After that, the experiment is repeated 5 times for each group with a constant speed of 100 mm/min and a preloading force of 1.0 N.

**Figure 3:**
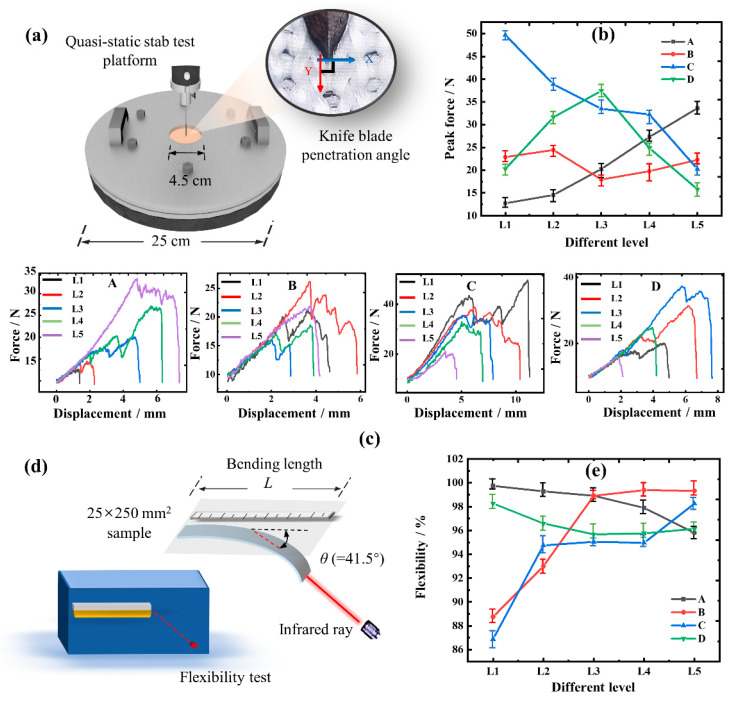
Single-factor analysis, and quasi-static stab and flexibility test. (**a**) Quasi-static stab test platform; (**b**) peak stab force of different levels; (**c**) stab force-displacement of different experimental schemes. A–D represent the force-displacement relationship for each of the four factor conditions; (**d**) flexibility test platform; (**e**) flexibility of different levels.

The quasi-static stab test of UHMWPE knitted fabric is carried out on the E43.504 MTS machine (MTS Systems (China) Co., Ltd., Shanghai, China) installing blade D1 in reference to ASTM D3787-2016 (2020) [40] and GA 68-2019 [41]. The direction *Y* of the blade when stabbed, is perpendicular to the horizontal *X* of the loop structure, as exhibited in Figure 3a. And, the size of the 10 × 10 mm^2^ sample is precisely held by a hollow disc and a bracket with an outer diameter of 25 cm and an inner diameter of 4.5 cm. Moreover, the force-displacement data of the stab resistance are obtained under the condition of a speed of 50 mm/min and a preloading force of 10.0 N.

The flexural rigidity of the fabric is measured to characterize the flexibility of the fabric referring to GB/T 18318-2009 [42]. According to Figure 3d, an automatic fabric stiffness meter (YG207, Ningbo Textile Instrument Factory, Ningbo, China) is used to detect the bending length of each 25 × 250 mm^2^ sample at the bending angle of 41.5°. And then, based on the Equation (1), the flexural rigidity of the fabric is calculated by the average bending length of each group. Next, the flexibility of the fabric can be obtained according to Equation (2).

(1) $$G=\frac{1}{tan\;\theta /cos\;\theta /2}\times \frac{PL^{3}}{8}\times 10^{-2}$$

(2) $$F=\frac{G}{G_{A}}\times 100\%$$

where *G* is the flexural rigidity of per unit width, mN cm; *θ* is an infrared angle, when *θ* = 41.5°, *G* ≈ *PL*^3^/8; *P* is the square meter weight of fabric, when *g* = 9.8, *P* = 9.8 the square meter quality of the fabric; *L* is the bending length, cm; *F* is the flexibility of the fabric; and *G_A_* is the flexural rigidity of per unit width in the case of unbending, that is. the flexural rigidity when *L* = 25.

## 3. Results and Discussion

### 3.1. Shear Resistant Force and Breaking Strength of UHMWPE

Figure 2b,d show the shear force-displacement curves and breaking strength-displacement curves of different yarn specifications, respectively. And, the same regular tendency is manifested in them, that is, the force gradually increases to the maximum value, and then decreases to a lower value. In Figure 2b, the S region is the shear breaking stage of UHMWPE. At the front of S region, the yarn was first straightened and extruded by the blade, as shown in Figure 2a. Indeed, the individual fiber that has been straightened can also be severed in this stage. Therefore, there exhibit multiple small peaks and fluctuations, and the length of the error bar in Figure 2e also appears longer. In addition, the shear force value reaches the maximum in the S section, but this does not mean the UHMWPE is completely cut. This is because most of the fibers simultaneously touch the blade and shear break, resulting in fracture failure. At this point, there is still a small amount of fiber that has not been cut and can continue to shear, so the curve of the S region is relatively gentle until the yarn has completely sheared failure. Moreover, the results show the peak shear force of the 200 D yarns, which are relatively thin, is only 2.49 N, which basically does not have the link of fluctuation and directly breaks. Besides, when the UHMWPE yarn thickens, the S region increases, which prolongs the shearing time of the yarn. After testing 1000 D UHMWPE yarn, the displacement of S region fluctuates about 0.7 mm, and the shear movement time of the yarn is nearly 2.1 s. Furthermore, compared to 200 D and 1000 D yarns, the UHMWPE peak shear force enhances from 2.49 N to 27.41 N, which increases the specific shear force by 3.32%, that is, the peak force that the yarn unit line density can withstand, N/tex.

When the UHMWPE yarn is stretched, it is gradually straightened and even undergoes elastic deformation. Then, the inner fiber of UHMWPE is pulled off successively when the displacement is more than 3 mm, it is shown as the ST region in Figure 2d. From the fact that the force value in the graph does not return to zero, because there are still some yarns that do not break. However, from the qualitative analysis, when the yarn is stretched by a certain displacement, the thicker the yarn, the greater the force value, and this leads to a larger breaking work. In other words, the area surrounded by the force-displacement curve is larger, which can also be seen from Figure 2e. Therefore, according to the above factors affecting the stab resistance of the knitted fabric, the different specifications of UHMWPE yarn are selected instead of the yarn shear resistance to carry out single factor analysis.

### 3.2. Analysis of Single-Factor

The single-factor method was conducted at four factors and five levels, as described in Table 1. Among them, the basic process conditions of this experiment were as follows: UHMWPE yarn specification is AL3, stitch density is BL3, fabric structure is CL3, and yarn blending ratio is DL1. Then, the significant effect of the influence factor on the stab-resistant effect and the better central value of the influence factor were obtained to carry out RSM analysis. Of course, the normality, difference, and variance homogeneity of one-way ANOVA experiments had also been verified to meet the requirements for use.

The statistical conclusion was analyzed at the 95% confidence level. If the *p*-value of a factor was less than 0.05, it was considered highly significant; otherwise, it was deemed not significant. And, the SPSS statistics 27.0.1 software was used to calculate the *p*-values of the normal test and the homogeneous test of the four groups factors. It showed they are greater than 0.05 in Table 1, which meant the samples show no significance, and the volatility of each sample data expressed consistency without a great difference. Then, further calculation results of ANOVA were obtained, where *p* of scheme B was 0.142 (*p* > 0.05), not showing a significant effect. In the other three groups, yarn size (A), structure (C), and blending ratio (D) had extremely significant effects on the stab-resistant performance of knitted fabric, C > A > D > B.

Meanwhile, the main parameters of each sample are summarized in Table 2. It is also evident from the table that the stitch density (B) and the blending ratio (D) exert the strongest influence on both the horizontal and longitudinal density of the material when compared with the other factors. Following that is the structural changes (C) of the fabric. These can impact the fabric’s properties by altering stitch length and fabric tightness. This finding reinforces the earlier analysis of loop deformation factors.

From Figure 3c(A), it is verified the yarn specifications are of great help to the enhancement of the stab-resistant performance of the fabric. Moreover, when the blade just started to stab the fabric, the force-displacement curve of the fabric with different yarn specifications basically overlaps, which is different from that of the B and C groups. It can be inferred the overlapping segment of the curves primarily occurs due to the deformation of the knitted loop structure, indicating the yarn specifications have no obvious influence on the loop deformation of the knitted structure. However, when the specification of UHMWPE yarn was increased from AL1 to AL5, the peak stab force of the fabric increased from 12.426 N to 34.758 N. In the meantime, the specific shear force of yarn was only increased by 3.32%, and the stab resistance of the fabric was improved by 179.72%, while the softness of the fabric was reduced by 3.99%. This result shows a significant impact of the improved yarn specification on the stab resistance of fabric, and highlights the importance of yarn’s shear resistance in optimizing the fabric stab resistance and flexibility.

Figure 3b,c(B) reveal a wave-like trend in the stab force curve of group B, where the force initially increases, then decreases before rising again with the increase of stitch density. This is due to the fact that, at a lower stitch density, the loop structures are relatively loose, allowing for greater deformation space. Therefore, when the blade is inserted, more energy is absorbed by the deformation of the fabric loop structure, which is more effective for stab resistance. This contradicts common sense, and the blade’s tip has penetrated the fabric at this point, causing harm to the human body. As a result, the effect of stitch density on the stab resistance of the fabric is not significant enough. Moreover, the change of fabric structure undoubtedly has an impact on the stab resistance of the fabric, whether it is knitted structure or woven structure. In Figure 3c(C), more resistances were created to the blade because of the complex interlocking relationship of loop structures within the fabric, thereby requiring more energy for the fabric to be completely punctured. This contributes to the fabric achieving stab resistance.

By changing the content of elastic yarn inside the fabric, the loop structure of the fabric has a certain elastic deformation, resulting in the improvement of the stab resistance. Hence, the blending ratio in group D also influences the loop deformation, as a method for stab resistance. However, different from the two groups of B and C, the force-displacement curves of BL1–BL5 remained basically consistent in the initial stage. This is because a change in the stitch density alters the size of the contact point loop, and the fabric structure modifies the form of the loop structure, both of which impact the loop’s stab resistance. Nevertheless, the variation in the blending ratio mainly affects the deformability of the loop structures while keeping the parameters of the loops in contact with the blade consistent. So, it does not affect the force mechanism of the loop structure, leading to the force values of BL1–BL5 in Group D following the same growth curve.

Figure 3e shows the flexibility of the test samples. In comparison to Figure 3b, the curve of the softness performance of the fabric is essentially opposite to its stab-resistant performance. Therefore, when designing for improved stab resistance, the factors such as fabric thickness, weight, and deformation are influenced, which may result in a decrease in its softness and flexibility. Also, this further reinforces the importance of optimizing the stab resistance and flexibility in the design of UHMWPE knitted structural fabrics.

### 3.3. Response Surface Method Optimization

Due to the non-significant data in the B group, the influence of three groups of A, B, and C factors on fabric stab resistance and flexibility was investigated, and the optimal process conditions were determined. Subsequently, the central composite design of the response surface was conducted, and the center values of AL3, CL3, and DL3 were selected, considering both stab resistance and flexibility, from Figure 3b,e.

By performing RSM calculations, a significant matched regression equation model, specifically a quadratic regression model, was obtained. This model helps to better fit the stab resistance and flexibility characteristics of the fabric. The formula is as follows:

(3) $$Y=\beta_{0}+\beta_{1}X_{1}+\beta_{2}X_{2}+\beta_{3}X_{3}+\beta_{12}X_{1}X_{2}+\beta_{13}X_{1}X_{3}+\beta_{23}X_{2}X_{3}+\beta_{11}{X_{1}}^{2}+\beta_{22}{X_{2}}^{2}+\beta_{33}{X_{3}}^{2}$$

where *Y* is the predicted response value; *X*_1_, *X*_2_, and *X*_3_ are the independent variables; *β*_0_ is the constant coefficient; *β*_1_, *β*_2_, and *β*_3_ are the first-order coefficients; *β*_12_, *β*_13_, *β*_23_ are the interaction coefficients; *β*_11_, *β*_22_, and *β*_33_ are the second-order coefficients. Besides, the specific values of the two predictive response formulas are shown in Table 3.

**Figure 4:**
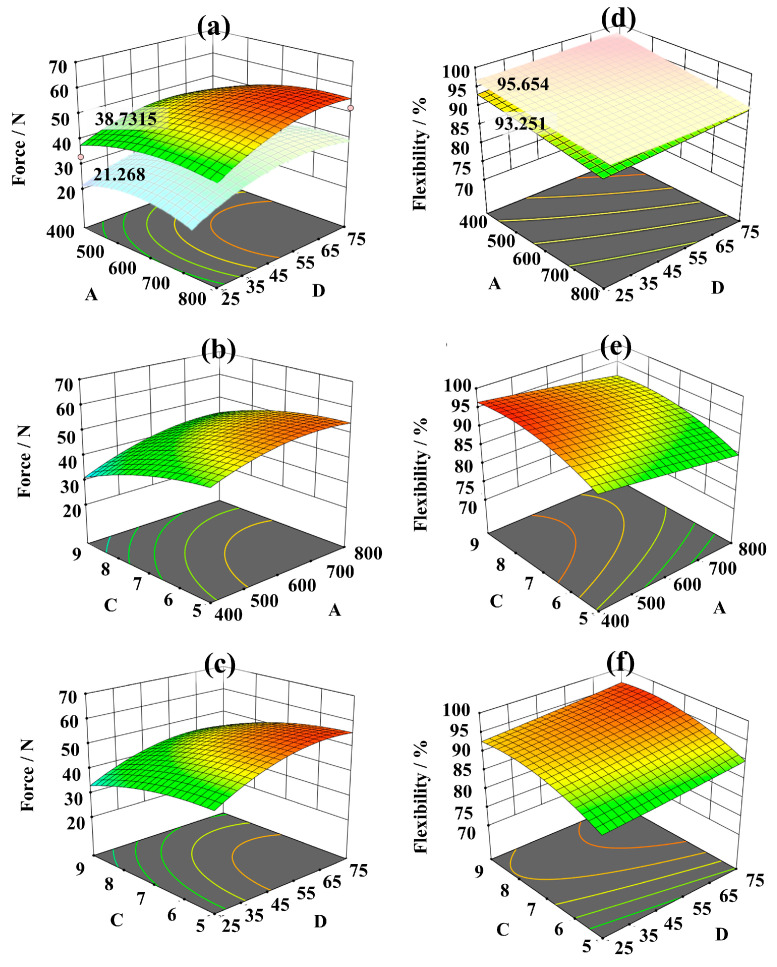
The response surface results of the three factors. (**a**–**c**) Stab-resistant response surface results of the three factors; (**d**–**f**) flexibility response surface results of the three factors. The sequence of blue–green–yellow–red in the graph indicates a gradual increase in performance. A, C, D denotes each of the three factors mentioned in Table 1.

Table 4 shows the stab resistance and flexibility results of the three factors, and then the response values of each factor are evaluated, as detailed in Table 5 and Table 6. And Figure 4 represent the calculated results of two response values for the three factors and the response surface results; respectively. It also expresses that factors A; C; and D; as well as A^2^; C^2^; and D^2^; are significant factors (*p* < 0.05) influencing the stab resistance performance of the fabric. Of particular note; factors C and D^2^ have the greatest impact on the stab resistance; with an effect of 0.0002. Beyond that; the interactions and mutual effects of other factors are not significant. Additionally; factors A; C; D; and C^2^ are statistically significant factors (*p* < 0.05) influencing the fabric’s flexibility. Among them; factor C^2^ has the most significant impact; with a value less than 0.0001. As a result; these three factors significantly affect both the stab resistance and flexibility of the fabric; with the range of the selected factor levels; the impact sequence of factors is C > A > D. This shows both models align well with the above single-factor experimental data. In Figure 4a,d; under the same levels of factors A and D; the peak force was increased from 21.268 N to 38.7315 N by altering the fabric’s structure; with an increase of 82.11%. While the flexibility only decreases from 95.654% to 93.251%, a decrease of only 2.51%, it indicates the importance of the knitted loop structure for flexible stab-resistant materials is prominent.

**Figure 5:**
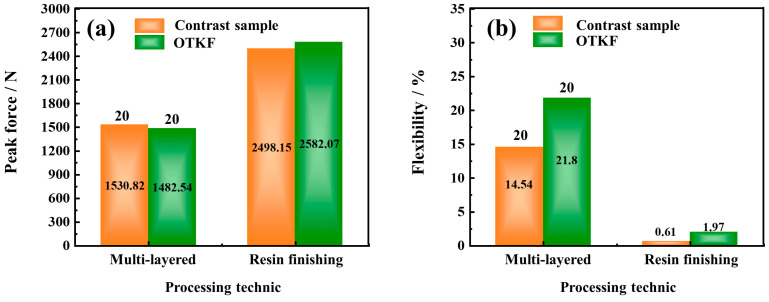
The performance comparison effect of the OTKF. (**a**) Peak force of the contrast experiment; (**b**) flexibility of the contrast experiment.

Then, the optimum process conditions of high stab resistance and flexibility fabric were determined by analysis. The values of factors A, C, and D are 640.799 D, 1 × 6.710 structure, and 67.999% content, respectively. In addition, the stab resistance and flexibility of the fabric were predicted to be 52.450 N and 93.6% under these conditions. To validate the feasibility of the predicted model, verification experiments were conducted using conditions drawn near the optimum. The peak stab force and flexibility of the fabric were measured as 54.016 N and 93.425%, which differed from the predicted values by only 2.33% and 0.187%, respectively. Moreover, the effect of the knitted fabric with the optimal technology (OTKF) was verified in Figure 5. The performance of the multi-layered stacked OTKF and the resin finishing OTKF was compared to the reference sample [43,44]. The preparation methods for these samples were based on the experimental steps described in the literature. Here, the materials for resin finishing OTKF and control samples were cured using E51 epoxy resin and amines hardener (Guangzhou Suixin Chemistry Co., Ltd., Guangzhou, China) with a ratio of 100:50, for 12 h at 25 °C. The density of the E51 epoxy resin is 1.18 g/cm³, the viscosity is 12,460 Mpa·s, and the epoxy equivalent is 186.2 g/ep. Due to differences in testing and preparation environments, it is reasonable to reveal some deviations between the reference values and the measured values of the comparison sample. Based on the results, it can be observed the OTKF has excellent peak force and flexibility. Although the OTKF exhibits a slight decrease in peak force, it still demonstrates more excellent flexibility and also achieves a satisfactory stab resistance (20 layers are not penetrated). Thus, it follows the process of optimizing fabric stab resistance and softness using the response surface model is indeed feasible.

## 4. Conclusions

To sum up, the knitted loop structure is prominent in the stab-resistant material. The analytical results indicate the stab resistance of the knitted fabric is mainly achieved by the loop deformation and the yarn shear resistance. Among them, the order of influence for the factors is as follows: fabric structure > yarn specification > yarn blending. The fabric structure exhibits the greatest impact on the stab resistance and flexibility of knitted fabrics, with a significance level of 0.0002. Specifically, through modifying the fabric structure, the peak force is strengthened by 82.11%, while the flexibility is reduced by 2.51%. Following that is the yarn specification; the shear resistance is improved with 3.32% by enhancing the yarn specification of UHMWPE (linear density range of 200–1000 D). This produces a significant increase of 179.72% in the quasi-static peak stab force of the fabric, while only reducing the softness performance by 3.99%. It further highlights the importance of yarn shear resistance in optimizing the stab resistance and flexibility of fabrics. Moreover, the influence of stitch density on peak force is negligible.

Furthermore, using the response surface methodology, the optimal process parameters for achieving excellent stab resistance and flexible performance were determined. Then, the knitted material exhibits a peak stab force of 52.45 N and a flexibility of 93.6% under the predicted process conditions, with only a 2.33% and 0.187% deviation from the actual test value. And, through the experimental comparison after retreatment, it is also verified this work can greatly optimize the stab resistance and flexibility of the textile matrix in flexible stab-resistant material and aid in the design and development of higher-quality stab-resistant garments.

## Figures and Tables

**Table 1:** Single-factor experiment and analysis of quasi-static stab results.

Factors		A: Yarn Specifications/(D)	B: Stitch Density	C: Fabric Structure	D: Blending Ratio/(%)
Levels	L1	200	−10	1 × 3	100
	L2	400	−5	1 × 5	75
	L3	600	0	1 × 7	50
	L4	800	5	1 × 9	25
	L5	1000	10	1 × 11	0
Normality	*p* (L1)	0.666	0.381	0.993	0.439
	*p* (L2)	0.761	0.176	0.630	0.209
	*p* (L3)	0.876	0.215	0.796	0.265
	*p* (L4)	0.676	0.263	0.867	0.672
	*p* (L5)	0.084	0.210	0.247	0.338
Homogeneity	*p*	0.287	0.986	0.547	0.932
ANOVA	F	14.930	2.052	8.894	6.008
	*p*	<0.001	0.142	<0.001	0.016

Note: The parameter of column A and row L1 in the table is denoted as AL1 in this experiment. And, the *p*-value and F-value are commonly used statistical indicators in ANOVA.

**Table 2:** Main parameters of each sample.

No.	Levels	Thickness/(mm)	Areal Density/(g·m^−2^)	Stitch Density /(Wales/5 cm × Courses/5 cm)	No.	Levels	Thickness/(mm)	Areal Density/(g·m^−2^)	Stitch Density /(Wales/5 cm × Courses/5 cm)
A	L1	0.69	168	70 × 118	C	L1	1.95	920	76 × 138
	L2	1.16	422	70 × 112		L2	1.87	777	70 × 124
	L3	1.47	504	64 × 106		L3	1.86	747	68 × 118
	L4	1.75	659	64 × 102		L4	1.87	733	68 × 115
	L5	1.97	771	60 × 100		L5	1.47	504	64 × 106
B	L1	1.41	677	74 × 192	D	L1	1.47	504	64 × 106
	L2	1.46	571	70 × 156		L2	1.51	528	64 × 118
	L3	1.47	463	68 × 140		L3	1.64	641	66 × 130
	L4	1.46	424	64 × 122		L4	1.65	711	66 × 136
	L5	1.49	367	64 × 106		L5	1.75	739	70 × 148

**Table 3:** The coefficient calculation results of the prediction model.

	*β*_0_	*β*_1_	*β*_2_	*β*_3_	*β*_12_	*β*_13_	*β*_23_	*β*_11_	*β*_22_	*β*_33_
Stab resistance	+49.9	+5.10	−6.32	+4.32	+1.10	+1.73	−0.6753	−3.72	−2.56	−5.00
Flexibility	+0.935	−0.027	+0.030	+0.0155	+0.0013	−0.0039	+0.0006	−0.0016	−0.026	+0.001

**Table 4:** Stab resistance and flexibility results of the three factors.

Experiment No.	A: Yarn Specifications/(D)	C: Fabric Structure	D: Blending Ratio/(%)	Peak Force/(N)	Flexibility/(%)
1	400	1 × 9	25	23.6227	94.1363
2	600	1 × 7	50	50.622	92.7015
3	600	1 × 7	100	39.6543	96.8728
4	600	1 × 7	50	53.01	92.4698
5	400	1 × 9	75	28.282	97.7589
6	600	1 × 3	50	58.306	74.4592
7	400	1 × 5	75	40.679	94.8173
8	1000	1 × 7	50	47.308	88.703
9	200	1 × 7	50	26.9573	96.9847
10	600	1 × 7	50	48.389	94.6545
11	600	1 × 7	0	24.303	90.907
12	400	1 × 5	25	33.054	90.4713
13	800	1 × 5	75	52.025	86.529
14	800	1 × 5	25	37.748	84.7117
15	600	1 × 7	50	46.025	95.7696
16	600	1 × 11	50	25.202	91.6756
17	800	1 × 9	25	32.4462	87.9252
18	600	1 × 7	50	49.763	93.5647
19	600	1 × 7	50	55.763	91.8816
20	800	1 × 9	75	44.2864	90.9779

**Table 5:** The calculated results of stab-resistant response values for the three factors.

	Sum of Squares	Degree of Freedom	Mean Square	F Value	*p* Value	Significant
Model	2232.10	9	248.01	13.14	0.0002	**
A	415.85	1	415.85	22.04	0.0008	**
C	638.53	1	638.53	33.84	0.0002	**
D	298.46	1	298.46	15.82	0.0026	**
AC	9.65	1	9.65	0.5116	0.4908	
AD	23.92	1	23.92	1.27	0.2865	
DC	3.65	1	3.65	0.1933	0.6695	
A^2^	347.08	1	347.08	18.39	0.0016	**
C^2^	164.79	1	164.79	8.73	0.0144	*
D^2^	629.56	1	629.56	33.36	0.0002	**
Residual	188.70	10	18.87			
Lack of Fit	129.71	5	25.94	2.20	0.2037	
Pure Error	58.98	5	11.80			

Note: * is significant (*p* < 0.05); ** is extremely significant (*p* < 0.01).

**Table 6:** The calculated results of flexibility response values for the three factors.

	Sum of Squares	Degree of Freedom	Mean Square	F Value	*p* Value	Significant
Model	0.0491	9	0.0055	12.49	0.0002	**
A	0.0119	1	0.0119	27.23	0.0004	**
C	0.0148	1	0.0148	33.97	0.0002	**
D	0.0038	1	0.0038	8.79	0.0142	*
AC	0.0000	1	0.0000	0.0319	0.8618	
AD	0.0001	1	0.0001	0.2750	0.6114	
DC	3.277 × 10^−6^	1	3.277 × 10^−6^	0.0075	0.9327	
A^2^	0.0001	1	0.0001	0.1433	0.7130	
C^2^	0.0170	1	0.0170	39.00	< 0.0001	**
D^2^	0.0000	1	0.0000	0.0621	0.8083	
Residual	0.0044	10	0.0004			
Lack of Fit	0.0033	5	0.0007	3.04	0.1240	
Pure Error	0.0011	5	0.0002			

Note: * is significant (*p* < 0.05); ** is extremely significant (*p* < 0.01).

## Data Availability

The data presented in this study are available upon request from the corresponding author.
